# The protective role of father behaviour in the relationship between maternal postnatal depression and child mental health

**DOI:** 10.1002/jcv2.12075

**Published:** 2022-05-03

**Authors:** Alex F. Martin, Barbara Maughan, Matt Jaquiery, Edward D. Barker

**Affiliations:** ^1^ Department of Psychology King's College London Institute of Psychiatry, Psychology & Neuroscience London UK; ^2^ Social, Genetic & Developmental Psychiatry Centre King's College London Institute of Psychiatry, Psychology and Neuroscience London UK; ^3^ Department of Experimental Psychology Medical Sciences Division University of Oxford Oxford UK

**Keywords:** ALSPAC, externalising, father‐child relationship, fathering, internalising, parental relationship, postnatal depression

## Abstract

**Background:**

Maternal depression, especially when severe and long‐lasting, is associated with adverse mental health outcomes in children. We aimed to assess, for children of mothers with persistent postnatal depression symptoms, whether positive father behaviours would decrease risk for conduct and emotional symptoms.

**Methods:**

Using data from 4009, mother–father–child trios from the Avon Longitudinal Study of Parents and Children we examined associations between maternal depression trajectories and positive father behavioural profiles across the postnatal period (child age: 2–21 months), and child conduct and emotional symptom trajectories across middle childhood (child age: 3.5–11 years).

**Results:**

Positive father behaviour was much less common in families where mothers experienced high‐persistent depression symptoms (33%) than in families where mothers did not (56%); of note, these fathers also had higher levels of depression symptoms. Using person‐level analysis, exposure to high‐persistent maternal depression symptoms increased child risk for a high trajectory of both conduct (odds ratio, 2.69; 95% CI: 2.00, 3.60) and emotional symptoms (odds ratio, 2.47; 95% CI: 1.83, 3.31). However, positive father behaviour (toward child and mother) reduced the odds of following high trajectories of conduct symptoms by 9% (*x* = 4.52, *p* < .001) and of emotional symptoms by 10% (*x* = 4.12, *p* < .001), even after controlling for father depression symptoms. Using variable‐level analysis, we did not identify an interaction between maternal depression and positive father behaviour. For conduct problems, we identified a direct effect of positive father behaviour and lower conduct problems. For emotional symptoms, father behaviour interacted with child age, where the largest decrease was seen at age 9, when symptoms were highest across the sample.

**Conclusions:**

Positive father behaviour can be protective against chronic mental health problems for children exposed to persistent maternal postnatal depression symptoms.

## Graphical abstract

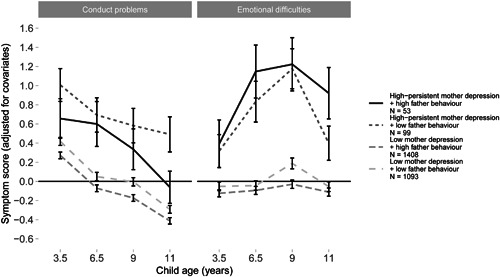

Maternal depression is associated with adverse mental health outcomes in children. We found that positive father behaviour, toward both the mother and the child during infancy, can attenuate this risk pathway.

**Key points**

**What's known:**

‐ Maternal postnatal depression symptoms are associated with adverse mental health outcomes in children.

**What's new:**

‐ We investigated how father behaviours, through parenting and partner support, may buffer this risk pathway.
‐ Positive father behaviour was less common in families where mothers experienced persistent depression symptoms, and fathers reported increased depression symptoms.
‐ Nonetheless, we found that consistent positive father behaviour offered some protection against chronic conduct and emotional symptoms.

**What's relevant (clinical practice):**

‐ When mothers experience postnatal depression, clinical interventions which support fathers to better support their children and their partners may optimise child mental health outcomes.

## Introduction

Around 1 in 10 women experience clinical levels of depression symptoms after the birth of a child (Howard et al., 2014). Postnatal depression is associated with increased risk of child behavioural and emotional problems, and that risk is most marked when depression symptoms are high and persist across the postnatal period (Netsi et al., 2018). But, mental health outcomes in children are not solely determined by the mental health of mothers; a number of other factors—including father's parenting and partner support—may buffer this risk pathway (Stein et al., 2014).

Although evidence on the potential buffering effects of positive father behaviours is limited, some studies have reported on the impact of both positive father–child relationships and father support to mothers. In relation to father–child relationships, Mezulis et al. (2004) prospectively examined maternal depression from birth to 12 months, and father‐reported parenting at 12 months. They identified a protective effect of positive father–child relationships on child emotional symptoms that lasted until the child was 4 years old. Chang et al. (2007) examined maternal depression at baseline (when children were aged between 0 and 10 years old) and child symptom trajectories between ages 4 and 14 years. They found a buffering effect of father involvement on this risk pathway, based on reports of fathering by the child at one time point, when they were aged 10. Tannenbaum and Forehand (1994) also found a protective effect of child‐reported father–child relationship, although this study was cross‐sectional and all measures, including maternal depression, were assessed when the child was 13 years old. Taken together, these findings suggest that when mothers are depressed, the father–child relationship can be protective, though the timing of these effects remains unclear.

As well as supporting their children, fathers can also provide emotional and social support to mothers when they are depressed (Holopainen, 2002). This may also provide a buffer for children by supporting the mothers through difficult periods. Evidence from clinical studies provides some insight. The Positive Parenting Programme (Triple‐P), for example, a well‐established intervention targeting child mental health outcomes via improvements in parenting, includes an additional component targeting positive partner support if mothers are depressed (enhanced Triple‐P: S. H. Goodman & Garber, 2017). A meta‐analysis of Triple‐P interventions identified small improvements in the parental relationship when families attended enhanced versus standard Triple‐P, along with improved child behavioural and emotional outcomes (Sanders et al., 2014). However, it is not clear, if these gains in child mental health outcomes were solely due to improvements in the parental relationship, or if there were also gains in father positivity toward the child.

To our knowledge, only one study has examined positive father behaviour toward both the child and mother in the same model. Mahedy et al. (2018) studied a group of high‐risk adolescents, whose mothers experienced severe and persistent depression. The authors examined father behaviours (reported by the mother and the child) when the child was in early adolescence, and child mental health 2 years later. Parental relationship quality was reported to increase with father emotional support to the child, and positivity toward both the child and the mother resulted in reduced adolescent depression symptoms at follow‐up.

While these findings are encouraging, it is important to bear in mind that father positivity toward the mother and/or the child may be less frequent in families where the mother experiences depression. Depression symptoms can co‐occur in mothers and fathers in the perinatal period (Paulson & Bazemore, 2010), though few studies have examined the extent to which the co‐occurrence of mother and father depression symptoms might impede positive father behaviour, or the impact this might have on children's mental health.

In this study, we examine data from a large epidemiological cohort to test prospective associations between severe and long‐lasting maternal depression and father behaviours during infancy, and child mental health symptoms across early and middle childhood. Based on the existing literature, our study had three aims. First, to examine associations between mother‐reported depression symptoms, father‐reported depression symptoms, and father‐reported positive behaviours toward the child and the mother, between infant ages 2–21 months. Second, to use a person‐centred approach to calculate the increased risk of children following a high trajectory of conduct and emotional symptoms (ages 3.5–11 years) when exposed to high levels of mother depression symptoms, and the reduction in risk based on positive father behaviour. Third, to use a variable‐centred approach to explore the long‐term stability of any protective effects of positive father behaviour when mothers experience depression symptoms in the postnatal period.

## METHODS

### Sample

Our study comprised participants from the Avon Longitudinal Study of Parents and Children (ALSPAC; Boyd et al., 2013; Fraser et al., 2013), an ongoing epidemiological study. Pregnant women resident in Avon, UK with expected dates of delivery 1st April 1991 to 31st December 1992 were invited to take part. The initial number of women enrolled was 14,541. These initial pregnancies resulted in 14,062 live births and 13,988 children alive at 1 year of age. The ALSPAC cohort is broadly representative of the general population in the UK. Please note that the study website contains details of all the data that is available through a fully searchable data dictionary and variable search tool http://www.bristol.ac.uk/alspac/researchers/our‐data/. Ethical approval for the study including informed consent was obtained from ALSPAC Law and Ethics Committee and the Local Research Ethics Committees http://www.bristol.ac.uk/alspac/researchers/research‐ethics/.

When surveyed 8 weeks after the birth of their child, 12,884 mothers had partners. Mothers were given the option to involve their partner in the study, of whom 8350 (65%) responded to the partner questionnaire at 8 weeks. Our sample included 4009 mother‐father‐child trios with complete father behaviour data; details are given below. Families included both biological and non‐biological, and resident and non‐resident fathers.

## MEASURES

### Depression symptoms

Depression symptoms in mothers were assessed using the Edinburgh Postnatal Depression Scale (EPDS), a widely used and validated 10‐item self‐report questionnaire (Cox et al., 1987). Assessments were at child ages 2, 8 and 21 months (*α*_s_ = 0.82–83). Scores range from 0 to 30, scores of 13 and above indicate high risk of perinatal depression (Levis et al., 2020). The EPDS is also validated for use in fathers (Ramchandani et al., 2008); father symptoms were assessed at child age 21 months (*α* = 0.82).

### Positive father behaviour

Fathers completed questionnaires relating to parenting and relationship attitudes and behaviours. Each behaviour was assessed once, at either 2, 8 or 21 months. Items were rated on 3–6 point Likert scales, recoded so that high scores denote more positive behaviours, summed into continuous subscale scores and standardised. Child‐focused items were taken from a standardised interview designed to assess the quality of parenting; details of scale construction are reported elsewhere (Dunn et al., 1999). Mother‐focused items were taken from the Dyadic Adjustment Scale, with subscale reliability ranging from *α* = 0.73 to 0.96 and strong content and concurrent validity (Fisher & Roberts, 2017). Subscales and items are reported in Table S1 in the Supporting Information S1.

*Child‐focused father behaviour:* Subscales related to the relationship with their child and attitudes to parenting: *Fatherhood Enjoyment* (*α* = 0.79), five items including ‘I enjoy the baby’ and ‘A baby has made me feel more fulfilled’; *Fatherhood Confidence* (*α* = 0.60), six items including ‘I feel confident with the baby’ and ‘I’m unsure whether I’m doing the right thing’. *Work and Parenthood* (*α* = .61), four items including ‘I enjoy getting home from work to see my partner and child’ and ‘After a day at work I find the baby hard to cope with’. Internal consistency was acceptable (*α* = 0.69).

*Mother‐focused father behaviour*: Subscales related to the relationship with and attitude to their partner: *Affection* (*α* = 0.89), 10 items assessing interactional affection including ‘Does your partner hug and kiss you’ and ‘Do you enjoy the company of your partner?‘; *Aggression* (*α* = 0.85), 3 items assessing interactional aggression including ‘Do you have arguments with your partner’ and ‘Does your partner get angry with you?‘; *Relationship Satisfaction* (*α* = 0.81), 7 items assessing satisfaction, including ‘feelings about sex’ and ‘feelings about making major decisions’. Internal consistency was acceptable (*α* = 0.69).

Factor analysis for each father behaviour subscale indicated that removing items did not increase reliability of the factor score.

### Child outcomes

Children's mental health difficulties were assessed using the conduct problems and emotional symptoms subscales of the Strengths and Difficulties Questionnaire (SDQ: Goodman, 1997). The assessment was completed by the mother when the child was aged 3.5, 6.5, 9 and 11 years (*α*_s_ = 0.61–0.68). Subscale scores range from 0 to 10. Thresholds associated with substantial risk of psychiatric disorder (Goodman, 2001) were established according to recommendations established from national data: conduct symptoms (scores of 4 and above), or emotional symptoms (scores of 5 and above).

### Covariates

Established correlates were controlled for in regression and ANCOVA analyses: child sex; father reported depression symptoms when the child was 8 months old; father non‐resident in the child's home at any postnatal timepoint; parent education (bachelor's degree or equivalent, or higher: coded as yes or no); family financial difficulty reported between 0 and 2 years old, taken from the Family Adversity Index: Bowen et al. (2005). The numbers of non‐biological fathers (14/4009) were too small for statistical analysis. Preliminary checks suggested no differences between biological and non‐biological fathers for all study variables, so biological and non‐biological fathers were not differentiated in the main analyses.

### Analytic sample and missing data

Missing data patterns for all study variables are reported in Figures S1–S3 (Supporting Information S1). Within our subsample, missing data were modelled using full information maximum‐likelihood (FIML), using a maximum likelihood parameter where standard errors are robust to nonnormality. FIML is recommended for longitudinal data with continuous and binary outcomes and is therefore most suitable for our analysis (Lim & Cheung, 2021). We only analysed families who had complete father data (*N* = 4247). Then, as our predictors and outcomes were to be assessed using latent profile analysis, those included in the analytic sample also had to include at least one data point for mother depression and child outcome for class membership to be estimated. Therefore, we also excluded those with no data for mother depression (1 excluded) and child outcomes (237 excluded).

The final analytic sample (*N* = 4009) was compared to those excluded (*N* = 10,492) using *t*‐tests and chi‐square tests. There was no significant difference observed in the proportion of female children between the analytic sample (female = 48.8%) and those excluded (female = 48.4%, *p* = .648). All other comparisons were significantly different (*p*s < .006). In the analytic sample compared to those excluded: depression scores were lower for mothers (*M* = 4.98 vs. 5.63) and fathers (*M* = 3.09 vs. 3.68); children had lower levels of conduct (*M* = 1.18 vs. 1.37) and emotional symptoms (*M* = 1.45 vs. 1.56); fathers were less likely to be non‐resident in the child's home (1.5% vs. 20.7%); parents were more likely to have a degree (14.2% vs. 8.1%); and families had lower levels of financial difficulty (11.1% vs. 18.9%). Of note, all variables were included in the analysis, which helps to decrease bias due to missing data (Little & Rubin, 2020).

### Statistical analysis

We examined father behaviour in two ways. First, we used latent profile analysis to isolate a high and consistent profile of positive father behaviour based on each behaviour subscale score. This father behaviour profile was examined in steps one and three (described below). Second, we explored mother‐focused and child‐focused father behaviour compared to a common factor comprising both, to determine which indicator to use in step two (described below).

The analysis proceeded in three main steps, with conduct and emotional symptoms as separate outcomes. In step 2, we examined child mental health outcomes at the person‐level by identifying a group of children with high and persistent levels of conduct and emotional symptoms. In step 3, we examined child mental health outcomes at the variable‐level, examining conduct and emotional symptom scores.

### Step 1: Associations between mother depression, positive father behaviour, and father depression

To isolate a high and persistent class of maternal depression symptoms which exceeded clinical threshold (13 and above: Levis et al., 2020) we used longitudinal latent profile analysis (LLPA), examining EPDS scores at child ages 2, 8 and 21 months (hereafter maternal depression trajectories). To isolate a high class of father behaviour, we used latent profile analysis (LPA), examining positive behaviour subscales reported at only one timepoint, between child ages 2–21 months (hereafter positive father behaviour profiles). A series of models were fitted, examining two‐, three‐ and four‐class models. To assess for indicators of bias, for each model we examined entropy, class separation, and scores at each timepoint, and evaluated the total cases in each trajectory or profile. We used this strategy because we were interested in assessing covariate effects (father behaviour) in a three‐step model. Entropy and class separation minimises bias in standard errors associated with covariates. We based this approach on recommendations by Heron et al. (2015).

We examined associations between trajectories of maternal depression symptoms, profiles of father behaviour, and father depression symptoms using chi square and *t*‐tests.

### Step 2: Person level analysis: The protective effect of positive father behaviour on high and persistent child mental health symptoms, when exposed to maternal depression symptoms

To examine child mental health symptoms at the person‐level, we used LLPA to identify a group of children with high and persistent trajectories of child conduct and emotional symptoms using SDQ subscale scores at child ages 3.5, 6.5, 9 and 11 years (following the approach described in step 1; hereafter conduct and emotional symptom trajectories).

To test whether father behaviour represented distinct dimensions (child‐focused and mother‐focused), or a common construct (child‐ and mother‐focused combined) we compared three latent factor structures (unidimensional; correlated factors; bifactor [one common and two specific residual factors]).

We tested whether exposure to a high maternal depression trajectory was associated with child conduct and emotional symptom trajectories using logistic regressions, controlling for the effect of father depression and other covariates. To assess the protective effect of father behaviour, we used an approach to quantify reduction in the odds of poor child outcomes after adding positive father behaviour factor/s to the regression model (OR_uncorrected_–OR_corrected_)/((OR_uncorrected_–1)*100%)), and tested the significance of the reduction using a Sobel test (MacKinnon et al., 2002).

### Step 3: Variable level analysis: Maternal depression, father behaviour and child mental health symptom scores across child ages

To examine child mental health symptoms at the variable‐level, that is, mean child mental health symptom scores, we used a repeated measures mixed ANCOVA, controlling for covariates as specified. Maternal depression group and father behaviour group were included as between subject variables, with child conduct and emotional symptom scores at each age as the within subject variables. We therefore test a three‐way interaction (maternal depression * positive father behaviour * conduct OR emotional symptoms).

Latent profile analysis and bifactor analysis were performed using Mplus version 8.2 (Muthén & Muthén, 2018). All other analyses were performed using R version 4.0.5 (R Core Team, 2021).

## RESULTS

Descriptives and correlations between study variables are shown in Table 1 (and stratified by maternal depression trajectory in Table S2 [Supporting Information S1]).

**TABLE 1:** Descriptive statistics and correlations for all study variables

	Mean/%	95% CIs	1	2	3	4	5	6	7	8
1. Maternal depression (high‐persistent trajectory)	5.9%	5.1, 6.6	‐							
2. Positive father behaviour (high profile)	54.9%	53.3, 56.5	−0.11***	‐						
3. Conduct symptom (high trajectory)	21.1%	19.8, 22.4	0.12***	−0.09***	‐					
4. Emotional symptom (high trajectory)	20.5%	19.3, 21.8	0.12***	−0.09***	0.24***	‐				
5. Child sex (male)	48.8%	47.3, 50.4	−0.02	−0.01	0.06***	−0.05***	‐			
6. Father depression score	3.09	2.99, 3.20	0.16***	−0.35***	0.09***	0.10***	−0.01	‐		
7. Non‐resident father (yes)	1.6%	1.2, 2.1	0.03	−0.04*	0.02	0.02	0.02	0.03*	‐	
8. Parent education (degree = yes)	14.2%	13.0, 15.3	0.02	−0.02	−0.05**	−0.05	−0.01	0.04*	−0.03	‐
9. Financial difficulties (yes)	11.1%	10.1, 12.1	0.13***	−0.05**	0.06***	0.05**	0.00	0.12***	0.04*	−0.03

*Note*: Pearson's and binomial correlations used as appropriate; CIs, confidence intervals.

Significance *p < .05, **p < .01, ***p < .001

### Step 1: Associations between maternal depression, positive father behaviour, and father depression

**FIGURE 1:**
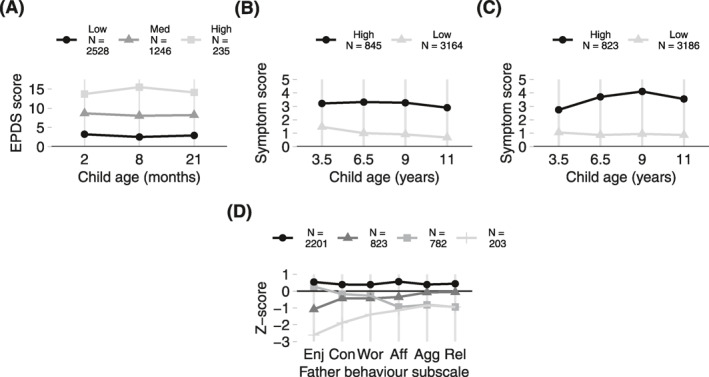
Latent profile analysis. (A) Maternal depression symptoms across the postnatal period, using the Edinburgh Postnatal Depression Scale; (B) Conduct symptoms across childhood, using the Conduct Problems subscale of the Strengths and Difficulties Questionnaire (SDQ); (C) Emotional symptoms across childhood, using the Emotional Difficulties subscale of the SDQ; (D) Positive father behaviour across subscales; Enj = Fatherhood Enjoyment; Con = Fatherhood Confidence; Wor = Work and Parenthood; Aff = Inter‐parental Affection; Agg = Inter‐parental Aggression; Rel = Relationship Satisfaction

*Mother postnatal depression trajectories*: A three‐class model provided the best fit to the data (Figure 1; steps and fit indices are in Table S3 and Figure S4 [Supporting Information S1]). Mothers in a ‘high‐persistent’ depression trajectory (6%) had EPDS scores of between ∼14 and 16 at each timepoint (scores above 13 indicate high‐risk of perinatal depression: Levis et al., 2020). Mothers in both the medium (31%) and low (63%) depression trajectories had stable scores well below this threshold, indicating consistently lower levels of depression symptoms. We therefore grouped together mothers in the medium and low trajectories (hereafter ‘low depression’, *N* = 3774), and compared them with those in the high‐persistent trajectory (*N* = 235).

*Father behaviour profiles*: A four‐class model produced the best fit to the data (Figure 1; steps and fit indices are in Table S3 and Figure S4 [Supporting Information S1]). The majority of fathers fell into a high‐positive behaviour profile (55%), with scores on all subscales (toward mother and child) at ∼0.5 standard deviations above the mean. In contrast, fathers in the other profile groups had lower scores and more variability across subscales, ranging between 0.1 and 2.6 standard deviations below the mean. We collapsed these groups (hereafter ‘low‐positive’, *N* = 1799) together, and compared them to the high‐positive profile (*N* = 2201).

Among mothers with high‐persistent depression, only 33% (*N* = 77) of fathers reported high‐positive behaviours, compared to 56% (*N* = 2124) of fathers in the remainder of the sample [*χ* ^2^(1) = 48.46, *p* < .001]. Fathers in this group also had significantly higher levels of depression symptom scores (*M* = 5.28, *SD* = 4.49) compared to those in the low maternal depression group [*M* = 2.96, *SD* = 3.33, *t*(250.27) = 7.79, *p* < .001, *d* = 0.68]. Father depression scores were also significantly higher in the low‐positive father behaviour profile (*M* = 4.41, *SD* = 3.89) compared to the high‐positive profile [*M* = 2.01, *SD* = 2.58, *t*(3021.5) = 22.48, *p* < .001, *d* = 0.74].

### Step 2: Person level analysis: The protective effect of positive father behaviour on high and persistent child mental health symptoms, when exposed to maternal depression symptoms

*Child symptom trajectories*: For conduct symptoms, a two‐class model produced a high and persistent trajectory (*N* = 845, 21%) and a low trajectory (*N* = 3164, 79%). For emotional symptoms, a two‐class model produced a high and persistent trajectory (*N* = 823, 21%) and a low trajectory (‘low’, *N* = 3186, 79%). Trajectories are presented in Figure 1, steps and fit indices are in Table S3 and Figure S4 (Supporting Information S1).

**FIGURE 2:**
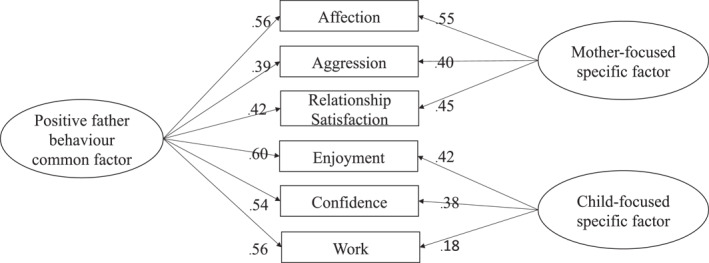
Bifactor model with standardised factor loadings: all loadings, *p* < .001

*Father behaviour toward child and mother*: A bifactor model of positive father behaviour represented the best fit to these data, compared to unidimensional and correlated two‐factor structures. The results are presented in full in Table S4 (Supporting Information S1). Additional bifactor‐specific analyses indicated that the specific factors (mother‐focused and child‐focused) did not provide a unique contribution to the total variance beyond that of the common father behaviour factor (Figure 2 and Table S5 [Supporting Information S1]). For the **common factor**: all subscales loaded significantly (range = 0.39–0.60), 62.4% of the variance in the total score was independently accounted for by the factor, and the *H* score (0.73) indicated good reliability. For the **specific factors**: the subscales loaded onto their respective subfactors, although all but two had smaller effects (range = 0.18–0.55). Having partitioned the variance for the common father factor, the specific factor indicator scores (*ω*_*H*_) did not reach threshold for clinical interpretation (> 0.50: Reise et al., 2013). The *H* score for both specific factors was also below reliability threshold. Therefore, the common positive father latent factor score, reflecting positive behaviour toward both the child and the mother, was extracted from the bifactor analysis and used in the following analysis. This standardised score represented individual differences in positive father behaviours toward the mother and child, with higher scores indicating higher levels of positivity (range −4.62 to 1.67).

As expected, after controlling for covariates, exposure to a trajectory of high‐persistent maternal depression symptoms was associated with increased odds of children following a high trajectory of both conduct symptoms (OR = 2.69; 95% CI: 2.00, 3.60) and emotional symptoms (OR = 2.47; 95% CI: 1.83, 3.31). Adding the common positive father behaviour factor to the regression model resulted in significant reductions in these odds: conduct symptoms, 8.88% reduction (*x* = 4.52, *SE* = 0.20 *p* < .001) and emotional symptoms, 9.52% reduction (*x* = 4.12, *SE* = 0.19, *p* < .001). The effects of maternal depression remained significant (Table 2; full table including all covariates is included in Table S6 [Supporting Information S1]).

**TABLE 2:** Logistic regressions and adjusted * odds ratios for a high trajectory of conduct and emotional symptoms between 3 and 11 years old, following exposure to high‐persistent maternal postnatal depression symptoms, with and without adjusting for positive father behaviour

	Conduct symptoms—high trajectory			Emotional symptoms—high trajectory		
** **	β	95% CI β	*p*	β	95% CI β	*p*
**Model 1: Maternal depression**						
Maternal depression (high persistent)	0.99	0.69, 1.28	<0.001	0.90	0.61, 1.20	<0.001
Odds ratio_adj_ [95% CIs]	2.69 [2.00, 3.60]^a^			2.47 [1.83, 3.31]^a^		
**Model 2: Maternal depression + father behaviour**						
Maternal depression (high persistent)	0.93	0.64, 1.23	<0.001	0.85	0.55, 1.14	<0.001
Common positive father factor^b^	−0.28	−0.40, −0.17	<0.001	−0.28	−0.39, −0.17	<0.001
Odds ratio_adj_ [95% CIs]	2.54 [1.87, 3.38]^c^			2.33 [1.73, 3.12]^c^		
OR reduction	8.88%			9.52%		

*Note*: * adjusted for child sex, father depression, non‐resident father status, parent education and financial difficulties; β, standardised beta; CI, confidence intervals.

^a^Maternal depression on child outcome.

^b^Continuous, standardised.

^c^Maternal depression on child outcome, adjusted for common father behaviour factor.

### Step 3: Variable level analysis: Maternal depression, father behaviour and child mental health symptom scores across child ages

**FIGURE 3:**
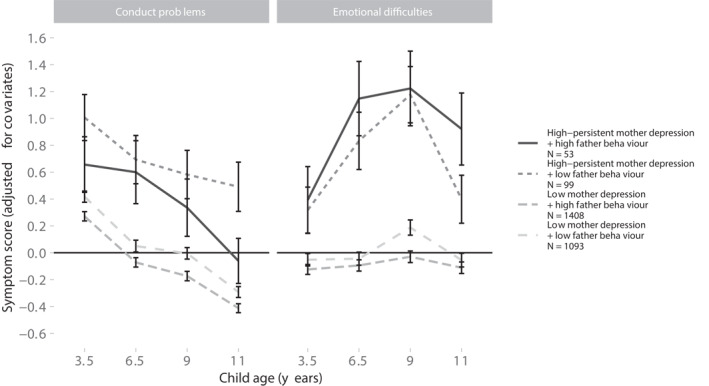
ANCOVA panel showing significant interactions and main effects for adjusted * conduct and emotional symptom scores. The within‐subjects predictor was child age (3.5, 6.5, 9, 11 years), and the between‐subjects predictors were father positive behaviour group (high vs. low) and mother depression group (high‐persistent vs. low); error bars are 95% confidence intervals; * adjusted for child sex, father depression, non‐resident father status, parent education, and financial difficulties

We used a repeated measures ANCOVA to investigate child conduct and emotional symptoms separately. The within‐subjects predictor was child age (3.5, 6.5, 9, 11 years), and the between‐subjects predictors were mother depression group (high‐persistent vs. low) and positive father behaviour group (high vs. low). ANCOVA results are reported in Figure 3 and Table S7 (Supporting Information S1).’

For child conduct problem scores, we did not observe any significant interactions between predictors. However, we identified three direct effects: 1. conduct symptoms were higher in the high‐persistent mother depression group (*F* = 39.05, *p* < .001); 2. conduct symptoms were lower in the high‐positive father behaviour group (*F* = 11.09, *p* < .001); and 3. conduct symptoms were highest at age 3.5 years and reduced at each timepoint, with the lowest levels reported at 11 years old (*F* = 245.81, *p* < .001).

For emotional difficulties, we identified an interaction of mother depression group and child age (*F* = 9.92, *p* < .001), and another interaction for positive father behaviour group and child age (*F* = 3.31, *p* = .020). Post hoc simple effects analysis identified significantly higher emotional symptoms at age 9 compared to all other ages (Bonferroni adjusted *p*s < .009). Regarding mother depression group * child age, symptoms were significantly higher in the high‐persistent depression group at each child age and mother depression group had the greatest effect at child ages 6.5 and 9 years (Bonferroni adjusted *p*s < .003). Regarding positive father behaviour group * child age, at age 9 symptoms were significantly lower when positive father behaviour was high (*m*_diff_ = 0.25, *t* = 3.63, *p* < .001). We did not observe a significant difference in symptoms between the positive father behaviour groups at any other child age.

## DISCUSSION

This study presents evidence that positive father behaviour can provide some protection against adverse mental health outcomes in children exposed to persistent maternal postnatal depression symptoms. Our results move forward understanding in three ways.

First, as expected, we identified that in families where mothers experienced persistent depression symptoms, fathers also reported higher levels of depression. In part, this may reflect previous findings suggesting that individuals who experience mental health difficulties tend to partner with those with similar psychopathology (Marmorstein et al., 2004). An equally plausible reason is the possibility of ‘contagion’ effects of depressive symptoms between partners (Joiner & Katz, 1999). Within the context of the birth of a child, it is important to note that both mother and father depression can peak in the first 6 months following birth (Paulson & Bazemore, 2010). When mothers experience depression symptoms during this early postnatal period, they can behave less positively toward their child (Lovejoy et al., 2000). We found that the same is true for fathers: in families where mothers experienced persistent depression symptoms, fathers reported fewer positive behaviours toward their children, but also toward their partners. Our findings suggest that depression symptoms in mothers and fathers correlate and within this context, fathers can show lower levels of positive behaviours during their child's earliest years.

Second, controlling for father depression symptoms, we found that at the person‐level, positive father behaviour toward both the child and the mother attenuated the relationship between maternal depression symptoms and high levels of child mental health difficulties. Why would positive father behaviour toward both act as a protective factor for the child? When mothers are depressed, fathers are often their primary source of social support (Holopainen, 2002), and when mothers are socially supported, this can improve their parenting. For example, father support has been found to minimise maternal over‐control and increase maternal sensitivity with their infants (Brunelli et al., 1995; Contreras, 2004). But what about the children? Of interest, when father positivity toward their child is high, family processes during early childhood, such as intra‐family warmth and cohesion, have been found to be unaffected by maternal postnatal depression (Vakrat et al., 2017). Our results extend these findings beyond infancy and early childhood, suggesting that even after accounting for the effect of father depression, consistent positive behaviour from the father during infancy can be protective against risk of both conduct and emotional symptoms across childhood.

Third, at the variable‐level, we did not identify an interaction between maternal depression and positive father behaviour. On the one hand, these findings suggest that when examining mean differences in child symptoms, positive father behaviour does not act as a protective factor within the specific context of maternal depression; rather, positive father behaviour, in general, relates to lower levels of conduct and emotional symptoms. However, this appears to contrast with the person‐centred analysis, which found that for a chronic trajectory of symptoms in children, father behaviour was protective following exposure to maternal depression symptoms. One potential explanation for this difference is that trajectory analysis (i.e. at the person‐level) assumes subgroups exist within a population, and that different trajectories associate with different aetiologies and outcomes (von Eye & Bogat, 2006). If positive father behaviour only has a protective effect in the context of maternal depression when child symptoms are high and chronic, this would not be identified when examining average levels of child symptoms (i.e. at the variable‐level), because the vast majority of children have low levels of symptoms. Several studies have indeed found associations with covariates in groups of children where symptoms are severe, but not at the variable‐level, for example, in relation to trauma and externalising psychopathology (Hagan et al., 2016).

In addition, Van Leeuwen et al. (2004) found a similar pattern of results to our study. They found that at the variable‐level, child personality and parenting scores independently associated with emotional symptoms. However, at the person‐level, the authors identified a group of children classed as ‘overcontrolled’ and a group of parents classed as ‘high negative control’, and these groups interacted: emotional symptoms were high when children were overcontrolled and highest when parents were also in the negative control group. The authors suggest that this difference was because person‐level analysis can reveal important associations for clinically at‐risk children, which can be missed at the variable‐level. This is equally likely in our study, especially considering that mental health symptoms in children can be most responsive to treatment when symptoms are both severe and chronic (Lorenzo‐Luaces et al., 2020). Therefore, a likely time that positive father behaviour can exert its effect is when mothers are persistently depressed, that is, when children are most at risk of high and chronic symptoms.

Our findings have clear implications for clinical practice. When mothers experience depression symptoms and positive father behaviour is low, intervening to help fathers improve their parenting and better support their partners may maximise gains in child mental health outcomes. This echoes findings from clinical studies: when mothers are depressed, interventions have been found to produce long‐term reductions in child mental health symptoms, especially when both parenting and partner support are targeted (Sanders et al., 2014). Our findings lend further weight to the importance of engaging fathers effectively in such interventions. Further, a commonality with studies which highlight discrepant person‐ versus variable‐level findings is their focus on clinical utility. In our study, our findings suggest that improving father support may be particularly important when child symptoms are at high levels. However, it is important to recognise complexity in risk pathways. For example, there is good evidence to suggest that treating maternal depression to remission also improves psychopathology symptoms in children (Weissman et al., 2006). Our findings point toward the value of including support for fathers in interventions, in addition to interventions tackling other risk pathways in families where mothers experience depression symptoms.

### Strengths and limitations

We used a longitudinal prospective cohort (ALSPAC) which is broadly representative of the UK population (Boyd et al., 2013). However, reliability for some subscales relating to father‐child behaviour was suboptimal (*α*_*s*_ = 0.60–0.68), and it will be important to test whether these scores replicate in a different sample, including whether it might be appropriate to examine the contribution of child‐ and mother‐focused father behaviours independently. ALSPAC provides assessments at multiple timepoints, enabling long‐term follow‐up and assessment of change over time. A particular strength of our analyses is the large population‐based sample. Despite this, like all longitudinal studies, ALSPAC faced problems of attrition. Specifically, mothers with persistent depression are known to be more likely to drop out of research studies (Netsi et al., 2018), and we found a similar pattern of drop out here. As a result, the proportion of women in the high‐persistent depression trajectory is likely to be underestimated in our analytic sample; we also found lower levels of child mental health symptoms in our sample compared to ALSPAC overall. However, within ALSPAC systematic drop‐out has been found not to alter the *associations* between family variables during pregnancy and child behavioural problems at age 8 (Wolke et al., 2009), broadly the same timeframe we explored in our longitudinal analysis. Overall, the validity of these simulation models was only marginally affected by participant drop‐out, but it resulted in more conservative estimates, suggesting that interpretation of our results may be similarly conservative. However, data loss in our sample may be additionally systematically impacted by mothers opting to include their partners and low levels of partner response.

## CONCLUSION

We found that in families where mothers experience high and persistent postnatal depression symptoms, fathers also had higher levels of depression symptoms, and were less likely to engage in positive fathering behaviours. In these families, children were at increased risk of following high trajectories of both conduct and emotional symptoms. However, if fathers reported consistently high‐positive behaviour toward both the child and the mother, this attenuated the association between maternal depression and child outcomes, even after accounting for the effect of father depression. Our findings highlight the importance of supporting fathers to support their children and their partners at the time when mothers are experiencing postnatal depression.

## CONFLICT OF INTEREST

Edward Barker is a member of the Editorial Advisory Board for JCPP *Advances*. The remaining authors have declared that they have no competing or potential conflicts of interest.

## ETHICS STATEMENT

Ethical approval for the study including informed consent was obtained from ALSPAC Law and Ethics Committee and the Local Research Ethics Committees http://www.bristol.ac.uk/alspac/researchers/research‐ethics/.

## AUTHOR CONTRIBUTIONS

**Alex Martin:** Conceptualization, Data curation, Formal analysis, Funding acquisition, Investigation, Methodology, Project administration, Supervision, Validation, Writing – original draft, Writing – review & editing. **Barbara Maughan:** Conceptualization, Investigation, Methodology, Supervision, Writing – review & editing. **Matt Jaquiery:** Formal analysis, Methodology, Writing – review & editing. **Ted Barker:** Conceptualization, Formal analysis, Funding acquisition, Investigation, Methodology, Supervision, Writing – review & editing.

## Supporting information

Supporting Information S1

## Data Availability

Data sharing is not applicable to this article as no new data were created or analysed in this study.
