# Supplementary material for: The protective role of father behaviour in the relationship between maternal postnatal depression and child mental health
Source: JCPP Adv. 2022 May 3;2(2):e12075. doi: 10.1002/jcv2.12075 (PMC10242879; doi:10.1002/jcv2.12075)
Supplement: Supplementary file 1 — Supporting Information S1 [file JCV2-2-e12075-s001.docx]

**The protective role of father behaviour in the relationship between maternal postnatal depression and child mental health**

Alex F. Martin, Barbara Maughan, Matt Jaquiery, Edward D. Barker

Table S1. Father behaviour factors, subscales and items

Figure S1. Number of cases lost due to missingness between the complete sample and the subsample for analysis

Figure S2. Proportion of missingness and combinations of missing data patterns within the complete sample for analysis for all father behaviour variables

Figure S3. Proportion of missingness and combinations of missing data patterns within the subsample for analysis for all study variables

Table S2a-c. Correlations for all study variables, and stratified by maternal depression trajectory

Table S3. Fit indices for maternal depression, father behaviour, and child mental health symptom latent profile models

Figure S4. Trajectory graphs showing 2- 3- and 4-class latent profile models for maternal depression, father behaviour, and child mental health symptoms

Table S4. Relative model fit information for estimated CFA models of father factors (child-focused and mother-focused)

Table S5. Model fit indices for bifactor model of father factors (common, child-focused and mother-focused)

Table S6. Logistic regressions and odds ratios for a high trajectory of conduct and emotional symptoms, following exposure to high-persistent maternal postnatal depression symptoms, with and without adjusting for positive father behaviour

Table S7

| Table S1: Father behaviour factors, subscales and items | | | |
| --- | --- | --- | --- |
| Subscale | Timepoint | Scoring | Item |
| **Mother-focused factor** | | | |
| Affection subscale | 8 months | Scoring: very often / often / sometimes / rarely / never | Would you say your partner is loving (affectionate) toward you? |
|  |  |  | Does your partner listen to you when you want to discuss your problems or talk about your feelings? |
|  |  |  | Does your partner talk to you about her problems and feelings? |
|  |  |  | Do you enjoy the company of your partner? |
|  |  |  | Does your partner show her approval of you? |
|  |  |  | Do you behave affectionately toward your partner? |
|  |  |  | Do you go out socially together? |
|  |  |  | Does your partner hug and kiss you? |
|  |  |  | Do you feel parenthood has brought you closer together? |
|  |  |  | Does your partner hold you in her arms? |
| Aggression subscale | 8 months | Scoring: very often / often / sometimes / rarely / never | Does your partner get angry with you? |
|  |  |  | Do you have arguments with your partner? |
|  |  |  | Do you get angry with your partner? |
| Relationship satisfaction | 1 year 9 months | Scoring: Very satisfied / moderately satisfied / somewhat dissatisfied / very dissatisfied | Feelings: Handling Family Finances |
|  |  |  | Feelings: Demonstrations of Affection |
|  |  |  | Feelings: Sex |
|  |  |  | Feelings: Time Spent Together |
|  |  |  | Feelings: Making Major Decisions |
|  |  |  | Feelings: Household Tasks |
|  |  |  | Feelings: Leisure Time & Activities |
| **Child-focused factor** | | | |
| Work and parenthood | 8 weeks | Scoring: always feel / often feel / sometimes feel / never feel | When I finish work I feel too tired to take the baby |
|  |  |  | I enjoy getting home from work to see my partner and child |
|  |  |  | When I finish work I take the child and let my partner get on with something she wants to do |
|  |  |  | After a day at work I find the baby hard to cope with |
| Fatherhood enjoyment | 8 months | Scoring: feel exactly / feel often / feel sometimes / never feel | I really enjoy the baby |
|  |  |  | It is a great pleasure to watch the baby develop |
|  |  |  | I feel I should be enjoying the baby but am not |
|  |  |  | A baby has made me feel more fulfilled |
|  |  |  | Babies are fun |
| Fatherhood confidence | 8 months | Scoring: feel exactly / feel often / feel sometimes / never feel | I would have preferred that we had not had this baby when we did |
|  |  |  | I feel confident with the baby |
|  |  |  | I dislike the mess that surrounds the baby |
|  |  |  | I really cannot bear it when the baby cries |
|  |  |  | I feel constantly unsure if I'm doing the right thing for the baby |
|  |  |  | I feel I have no time to myself |

Figure S1. Number of cases lost due to missingness between the complete sample and the subsample for analysis

Note. Missing data approach:

FIML is recommended for longitudinal data with a combination of continuous and binary variables, making it the appropriate solution for our data (e.g., Lim et al., 2021). Therefore, we applied FIML and associated steps to our analysis.

We assessed proportions and patterns of missingness within the data to assess whether we had to use complete-case analysis or where we could use FIML.

First, father behaviour had considerable missing data in the whole sample, with 71% missing at least one father behaviour variable and more than 50% were missing all father behaviour variables. Missing data patterns are now included in the supplementary materials. It is difficult to account for missing data using multiple imputation or maximum likelihood with levels missing >50% (Little & Robin, 2020); therefore, we initially restricted our sample to those with complete father data (N=4247) but allowed missingness on all other variables, thereby decreasing potential bias.

Second, those included in the analytic sample also had to include at least one data point for mother depression and child outcome for class membership to be estimated. Therefore, we restricted the sample to those with complete father data and at least one data point for mother depression (1 case excluded) and child outcomes (237 cases excluded). This gave a final sample of 4009 families.

Third, we reviewed missingness for all study variables within the subsample of 4009 families. All study variables had missingness <20% and were included in the analysis, except for IQ (over 50% missing), which we excluded.

REFERENCES. Lim, A. J. M., & Cheung, M. W. L. (2021). Evaluating FIML and multiple imputation in joint ordinal-continuous measurements models with missing data. Behavior Research Methods, 1. https://doi.org/10.3758/s13428-021-01582-w

Figure S2. Proportion of missingness and combinations of missing data patterns within the complete sample for analysis for all father behaviour variables

Note. L-R = affection, aggression, relationship satisfaction, work and baby, enjoyment, confidence

Figure S3. Proportion of missingness and combinations of missing data patterns within the subsample for analysis for all study variables

Note. childOC = child outcome (conduct and emotional symptom scores, missing at least one timepoint); highEdu = highest level of parent education; motherDp = mother depression missing at least one timepoint; nonRes = father residency status; fatherDp = father depression; childSex = sex of child

Table S2a. Correlations for all study variables in the high-persistent maternal depression group


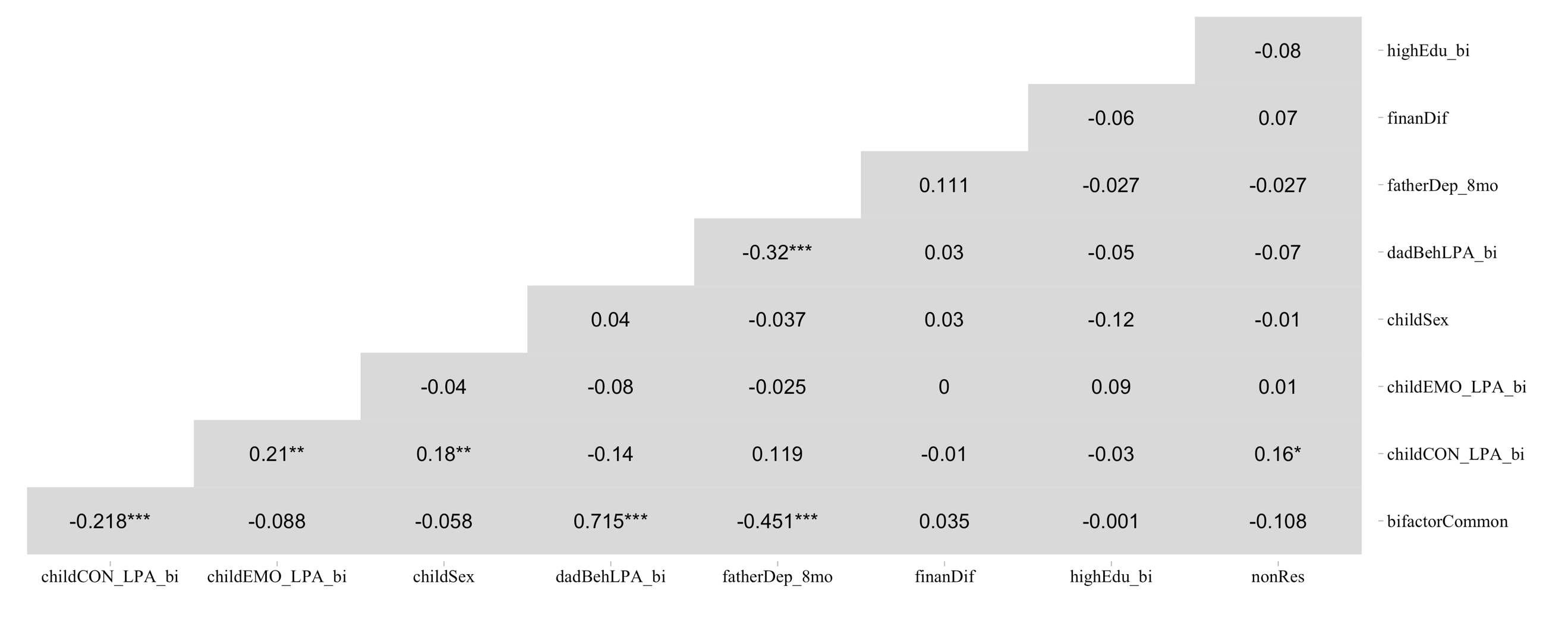


Table S2b. Correlations for all study variables in the low maternal depression group


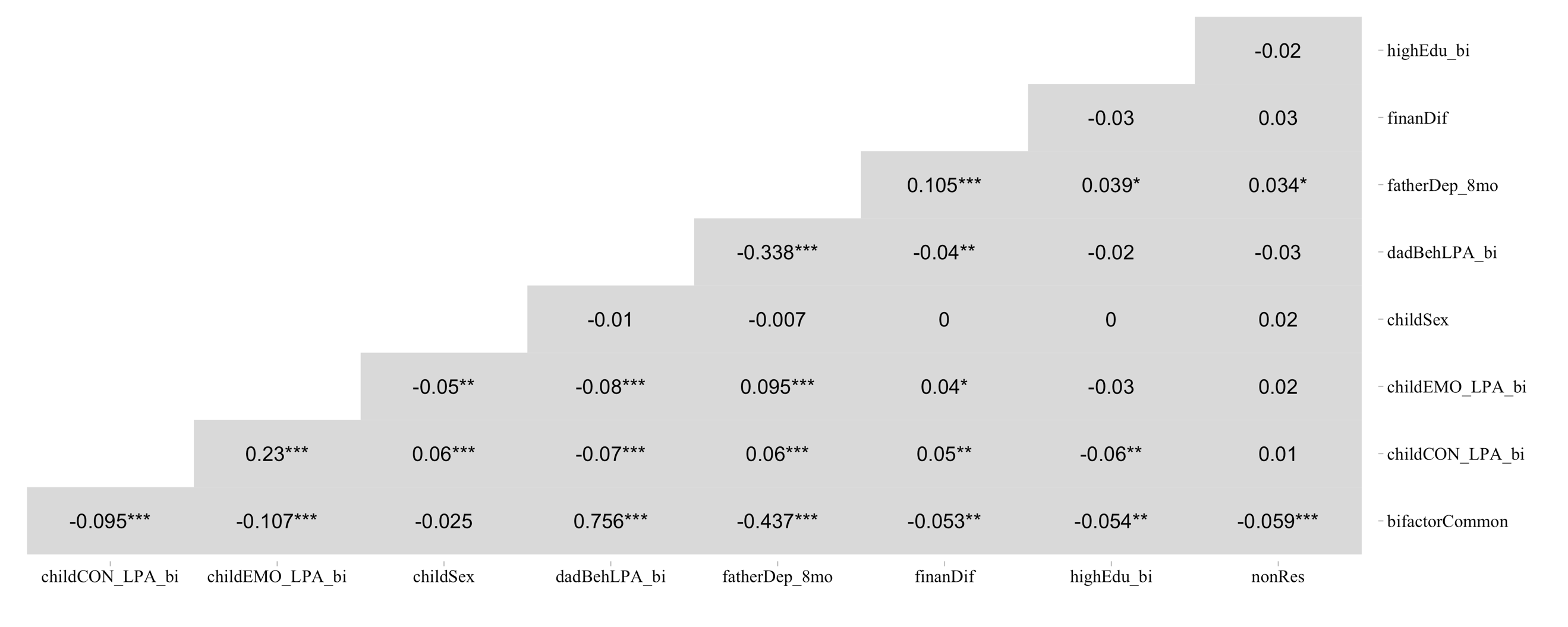


Table S2c. Correlations for all study variables in the study subsample, including biological father (which is not included in the final model)


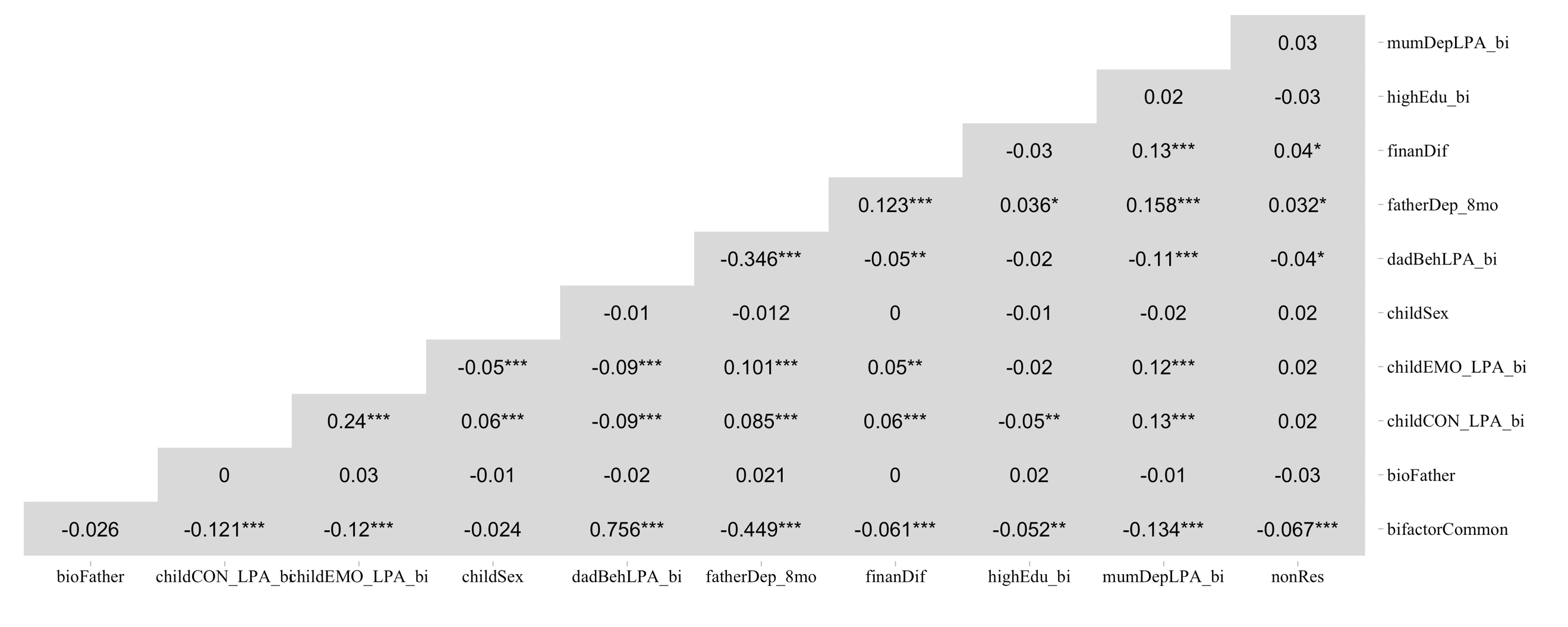


Note. Pearson's and binomial correlations used as appropriate; Significance *p < .05, **p < .01, ***p < .001; childCON_LPA_bi = child conduct symptom trajectory; childEMO_LPA_bi = child emotional symptom trajectory; childSex = sex of child; dadBehLPA_bi = father behaviour profile; fatherDep8mo = father depression score at 8 months; highEdu_bi = highest level of parent eduction, bachelors degree yes/no; mumDepLPA_bi = maternal depression trajectory; nonRes = father residency status

| Table S3. Fit indices for maternal depression, father behaviour, and child mental health symptom latent profile models | | | | | | | |
| --- | --- | --- | --- | --- | --- | --- | --- |
|  | AIC | BIC* | Entropy | BLRT | *p* value of BLRT | N high group | % high group |
|  |  |  |  |  |  |  |  |
| **Maternal postnatal depression** | |  |  |  |  |  |  |
| 2 class model | 65360.4 | 65391.6 | 0.834 | 3618.5 | < .001 | 994 | 24.79% |
| 3 class model | 64324.9 | 64368.5 | 0.829 | 1043.5 | < .001 | 235 | 5.86% |
| 4 class model | 64085.3 | 64141.5 | 0.829 | 247.5 | < .001 | 218 | 5.44% |
| **Positive father behaviour** |  |  |  |  |  |  |  |
| 2 class model | 64819.9 | 64879.1 | 0.766 | 3474.4 | < .001 | 2943 | 73.41% |
| 3 class model | 63593.1 | 63674.2 | 0.744 | 1240.8 | < .001 | 2221 | 55.40% |
| 4 class model | 62868.7 | 62971.6 | 0.791 | 738.40 | < .001 | 2201 | 54.90% |
| **Conduct problems** |  |  |  |  |  |  |  |
| 2 class model | 45185.6 | 45226.2 | 0.821 | 3924.7 | < .001 | 845 | 21.08% |
| 3 class model | 43868.8 | 43924.9 | 0.812 | 1326.8 | < .001 | 191 | 4.76% |
| 4 class model | 43493.8 | 43565.5 | 0.753 | 385.0 | < .001 | 113 | 2.82% |
| **Emotional difficulties** |  |  |  |  |  |  |  |
| 2 class model | 49736.4 | 49777.0 | 0.851 | 4075.9 | < .001 | 823 | 20.53% |
| 3 class model | 48799.5 | 48855.6 | 0.838 | 947.0 | < .001 | 186 | 4.64% |
| 4 class model | 48459.4 | 48531.2 | 0.826 | 350.0 | < .001 | 192 | 4.79% |

Note. * Sample size adjusted.

For the longitudinal symptom trajectories (mother depression, child conduct symptoms, child emotional symptoms), we assessed entropy, evaluated separation between classes, and examined numbers and proportions in each class to ensure sufficient cases in each class for analysis. We used this strategy because we were interested in assessing covariate effects (father behaviour) in a three-step model, therefore it was important to ensure that the standard errors were precise (i.e., low in bias). We based this approach on recommendations by Heron, Croudace, Barker and Tilling (2015), who tested simulations and epidemiological data and reported that low class separation (i.e. latent classes that either cross or are close in means) can result in biased standard errors, even when entropy is high (> .70). To note, for our research question, we were primarily interested in examining a high and persistent class of symptoms to be compared to non-high/less persistent classes.

We do not report 5-class models or above as either the models failed to converge, entropy was too low, or there was insufficient class separation between the high class and other classes in the model.

For maternal depression trajectories, we selected the 3-class model as it had the highest entropy, clear class separation, as well as a sufficient group size for analysis, and symptoms tracked the clinical threshold (13 and above) at each timepoint.

For conduct and emotional symptom trajectories, we selected the 2-class models as they had the highest entropy, clear class separation, high symptoms at each timepoint, and left a sufficient group size for analysis.

For father behaviour profiles, the 2- 3- and 4-class models all produced a clear class separation for the high class with scores well above the mean for all subscales. We selected the 4-class model as it had the highest entropy which for this analysis, was also supported by the lowest bootstrapped LRT. The high class had scores ~0.5 sd above the mean for all subscales. The other three classes all had scores below the mean for each subscale, and although there was low separation between them, we collapsed them to enable a high versus low father behaviour comparison.

REFERENCES. Heron, J., Croudace, T. J., Barker, E. D., & Tilling, K. (2015). A comparison of approaches for assessing covariate effects in latent class analysis. Longitudinal and Life Course Studies, 6(4), 420–434.


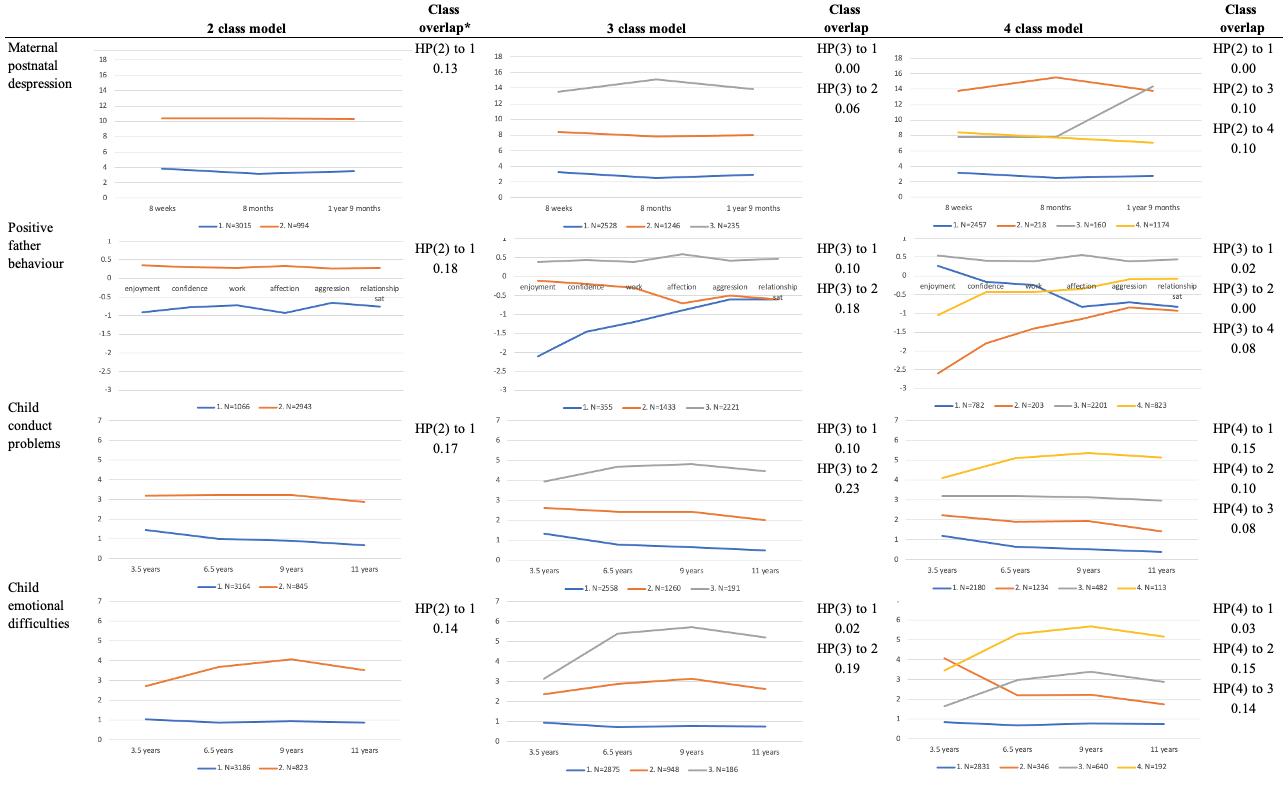


Figure S4. Trajectory graphs showing 2- 3- and 4-class latent profile models for maternal depression, father behaviour, and child mental health symptoms

Note. * Class overlap is calculated by summing the classification probabilities for most likely class membership, a score closer to zero indicates less class overlap, we assessed the high-persistent trajectory/profile to each of the other trajectories/profiles in the model; HP = high-persistent

| Table S4. Relative model fit information for estimated CFA models of father factors (child-focused and mother-focused) | | | | |
| --- | --- | --- | --- | --- |
|  |  |  |  |  |
|  | CFI | TLI | RMSEA | AIC |
| Bifactor | 0.98 | 0.91 | 0.08 | 111899.0 |
| Two factors (correlated) | 0.97 | 0.91 | 0.06 | 111906.5 |
| Unidimensional | 0.78 | 0.64 | 0.16 | 112650.3 |

Note. CFI = comparative fit index (good fit) ≥.95); TLI = Tucker-Lewis index (good fit) ≥.95); RMSEA = root mean square error of approximation (close fit ≤.05, acceptable fit ≤.08); CIs = confidence intervals; AIC = Akaike information criteria

Model fitting father behaviour

Using Mplus v8.2 (Muthén & Muthén, 2018), we compared three latent factor structures for positive father behaviour toward the child (3 items) and the mother (3 items), following the steps described in Meehan et al. (2018): 1) unidimensional (one factor), 2) two correlated factors, 3) bifactor (one general, two specific, shared variance between the factors fixed to zero; Brown, 2006). Relative fit was assessed using the comparative fit index (CFI), scores above .95 indicate good fit; the Tucker-Lewis index (TLI), scores above .95 indicate good fit (Hu & Bentler, 1999); the root mean square error of approximation (RMSEA), scores below .08 indicate acceptable fit and below .05 indicates good fit (Brown & Cudeck, 1993); the Akaike information criterion (AIC; lower scores indicate better fit). All fit parameters and model comparison indices are reported in table S3.

In order to test whether the two specific factors (child- and mother-focused) represent meaningful constructs above and beyond the common factor, or whether the model was over-fitted, the bifactor model was then tested against reliability metrics (Rodriguez, Reise, & Haviland, 2016): 1) omega statistic (ω), an estimate of the proportion of variance in the total score attributed to all modelled factors (i.e., common factor and both specific factors); 2) omega subscale statistic (ωS), an estimate of the proportion of variance for each subscale total score, attributed to the common variance of the common factor and subscale score; 3) omega hierarchical statistic (ωH), an estimate of the proportion of unique variance in the subscale score.

Bifactor analyses indicated a common factor of positive father behaviour, and not the specific factors (mother-focused and child-focused), represented the best fit to the data (Figure 2 and Table S4). For the common factor: all subscales loaded significantly (range = .39-.60), 62.4% of the variance in the total score was independently accounted for by the factor, and the H score (.73) indicated good reliability. For the specific factors: the subscales loaded onto their respective subfactors, although all but two had smaller effects (range = .18-.55). Having partitioned the variance for the common father factor, the specific factor indicator scores (ωH) did not reach threshold for clinical interpretation (>.50; (Reise, Bonifay, & Haviland, 2013). The H score for both specific factors was also below reliability threshold.

Therefore, the common positive father latent factor score, reflecting positive behaviour toward both the child and the mother, was extracted from the bifactor analysis and used in the ensuing analysis.

REFERENCES. Brown, T. A. (2006). Confirmatory factor analysis for applied research. New York: Guildford Press.

Browne, M. W., & Cudeck, R. (1993). Alternative ways of assessing model fit. In K. A. Bollen & J. Long (Eds.), Testing Structural Equation Models (pp. 136–162). Newbury Park, CA: Sage.

Hu, L. T., & Bentler, P. M. (1999). Cutoff criteria for fit indexes in covariance structure analysis: Conventional criteria versus new alternatives. Structural Equation Modeling, 6(1), 1–55. https://doi.org/10.1080/10705519909540118

Meehan, A. J., Hawes, D., Salekin, R. T., & Barker, E. D. (2018). Shared and unique variances of interpersonal callousness and low prosocial behavior. Psychological Assessment. Psychological Assessment. https://doi.org/10.1016/j.pec.2013.10.008

Muthén, L. K., & Muthén, B. O. (2018). Mplus User’s Guide. Eighth Edition. (1998-2017). Los Angeles, CA: Muthén & Muthén.

Reise, S. P., Bonifay, W. E., & Haviland, M. G. (2013). Scoring and modeling psychological measures in the presence of multidimensionality. Journal of Personality Assessment, 95(2), 129–140. https://doi.org/10.1080/00223891.2012.725437

Rodriguez, A., Reise, S. P., & Haviland, M. G. (2016). Evaluating bifactor models: Calculating and interpreting statistical indices. Psychological Methods, 21(2), 137–150.

Vrieze, S. I. (2012), Model selection and psychological theory: a discussion of the differences between the Akaike Information Criterion (AIC) and the Bayesian Information Criterion (BIC). Psychological Methods, 17(2), 228–243.https://doi.org/10.1037/a0027127

| Table S5. Model fit indices for bifactor model of father factors (common, child-focused and mother-focused) | | | | | | | |
| --- | --- | --- | --- | --- | --- | --- | --- |
|  | Bifactor-derived statistic | |  |  |  |  |  |
|  | ω / ω_S_ | ω_H_ / ω_HS_ | Relative ω | Correlation | H | ECV | PUC |
| General | 0.792 | 0.624 | 78.8% | 0.79 | 0.727 | 0.643 | 0.600 |
| Child-factor | 0.710 | 0.076 | 10.7% | 0.28 | 0.184 |  |  |
| Mother-factor | 0.696 | 0.405 | 58.2% | 0.64 | 0.511 |  |  |

Note. ω = omega (for common factor); ωS = omega subscale (for child and mother factors); ωH = omega hierarchical (for common factor); ωHS = omega hierarchical subscale (for child and mother factors); relative ω = proportion of variance in the common/subscale factor due to the common/subscale factors; correlation = correlation between factor and observed total score; H = construct replicability; ECV = explained common variance; PUC = percentage uncontaminated correlations


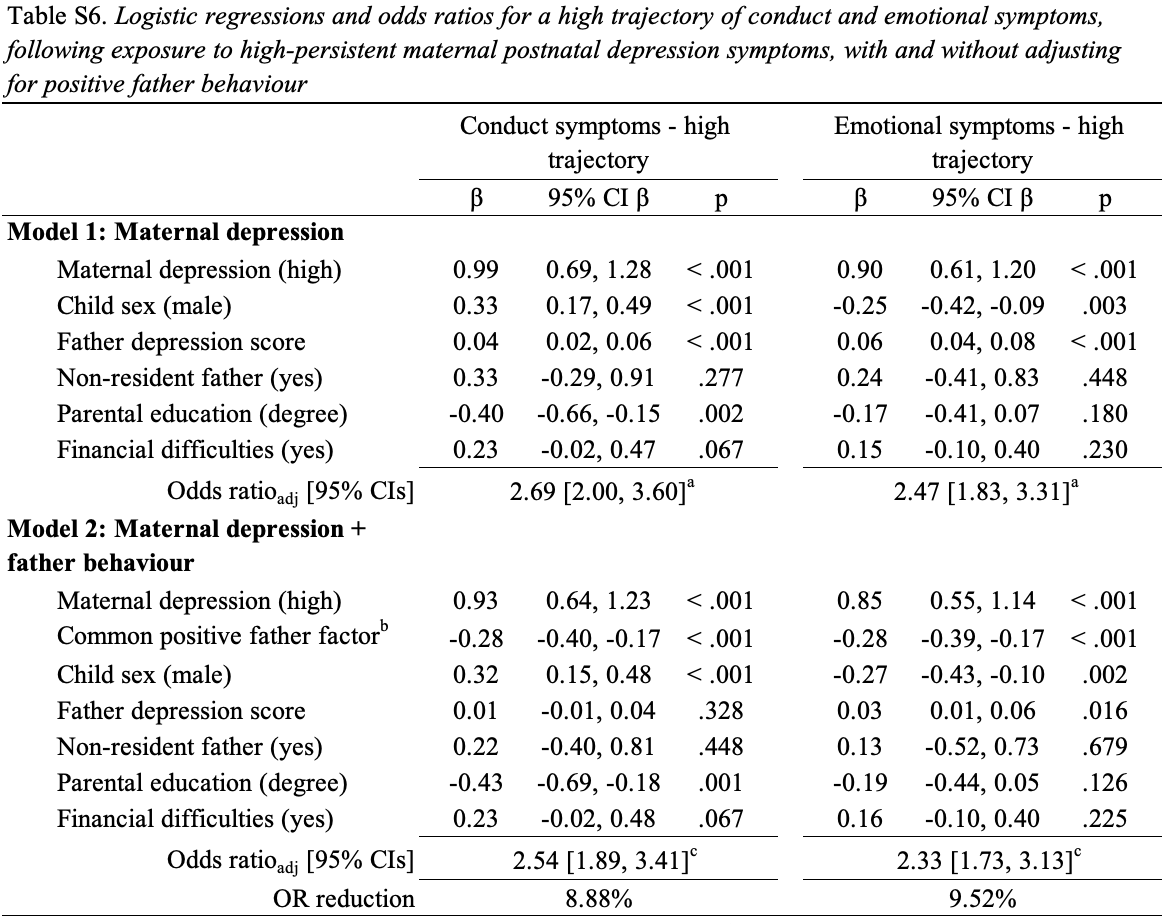


Note. β = standardised beta; CI = confidence intervals; p = p value; a maternal depression on child outcome; b continuous, standardised; c maternal depression on child outcome, adjusted for common father behaviour factor

| Table S7. R*epeated measures ANCOVA* testing the effects of within-subjects child age (3.5, 6.5, 9, 11 years), between-subjects group (maternal depression high-persistent/low), and between-subjects group father positive behaviour high/low) on child mental health symptom scores (conduct OR emotional symptoms)* | | | | | | | |
| --- | --- | --- | --- | --- | --- | --- | --- |
|  |  |  |  |  |  |  |  |
|  |  |  | Conduct symptoms | |  | Emotional symptoms | |
|  |  |  |  |  |  |  |  |
|  | *df* |  | *F* | *p* |  | *F* | *p* |
| Mother depression * father behaviour * child age | 3, 7947 |  | 1.46 | .224 |  | 0.81 | .485 |
| Father behaviour * child age | 3, 7947 |  | 0.38 | .761 |  | 3.31 | .020 |
| Mother depression * child age | 3, 7947 |  | 0.65 | .575 |  | 9.92 | < .001 |
| Mother depression * father behaviour | 1, 2649 |  | 0.83 | .362 |  | 2.30 | .129 |
| Child age | 3, 7947 |  | 245.81 | < .001 |  | 15.61 | < .001 |
| Father behaviour (high) | 1, 2649 |  | 11.09 | < .001 |  | 2.28 | .131 |
| Mother depression (high-persistent) | 1, 2649 |  | 39.05 | < .001 |  | 53.05 | < .001 |

Note. * adjusting for child sex, father depression, non-resident father status, parent education and financial difficulties; df = degrees of freedom; p = p value (corrected for sphericity where required)
